# A computerized tomography-based radiomic model for assessing the invasiveness of lung adenocarcinoma manifesting as ground-glass opacity nodules

**DOI:** 10.1186/s12931-022-02016-7

**Published:** 2022-04-16

**Authors:** Minghui Zhu, Zhen Yang, Miaoyu Wang, Wei Zhao, Qiang Zhu, Wenjia Shi, Hang Yu, Zhixin Liang, Liangan Chen

**Affiliations:** 1 Chinese People’s Liberation Army Medical School, Beijing, 100853 China; 2 Department of Respiratory Medicine, First Medical Center of Chinese People’s Liberation Army General Hospital, Beijing, 100853 China; 3 Department of Pulmonary and Critical Care Medicine, Zhongnan Hospital of Wuhan University, Wuhan, 430071 China

**Keywords:** Radiomics, Computerized tomography, Lung adenocarcinoma, Ground-glass opacity nodules, Invasiveness

## Abstract

**Background:**

Clinically differentiating preinvasive lesions (atypical adenomatous hyperplasia, AAH and adenocarcinoma in situ, AIS) from invasive lesions (minimally invasive adenocarcinomas, MIA and invasive adenocarcinoma, IA) manifesting as ground-glass opacity nodules (GGOs) is difficult due to overlap of morphological features. Hence, the current study was performed to explore the diagnostic efficiency of radiomics in assessing the invasiveness of lung adenocarcinoma manifesting as GGOs.

**Methods:**

A total of 1018 GGOs pathologically confirmed as lung adenocarcinoma were enrolled in this retrospective study and were randomly divided into a training set (n = 712) and validation set (n = 306). The nodules were delineated manually and 2446 intra-nodular and peri-nodular radiomic features were extracted. Univariate analysis and least absolute shrinkage and selection operator (LASSO) were used for feature selection. Clinical and semantic computerized tomography (CT) feature model, radiomic model and a combined nomogram were constructed and compared. Decision curve analysis (DCA) was used to evaluate the clinical value of the established nomogram.

**Results:**

16 radiomic features were selected and used for model construction. The radiomic model exhibited significantly better performance (AUC = 0.828) comparing to the clinical-semantic model (AUC = 0.746). Further analysis revealed that peri-nodular radiomic features were useful in differentiating between preinvasive and invasive lung adenocarcinomas appearing as GGOs with an AUC of 0.808. A nomogram based on lobulation sign and radiomic features showed the best performance (AUC = 0.835), and was found to have potential clinical value in assessing nodule invasiveness.

**Conclusions:**

Radiomic model based on both intra-nodular and peri-nodular features showed good performance in differentiating between preinvasive lung adenocarcinoma lesions and invasive ones appearing as GGOs, and a nomogram based on clinical, semantic and radiomic features could provide clinicians with added information in nodule management and preoperative evaluation.

**Supplementary Information:**

The online version contains supplementary material available at 10.1186/s12931-022-02016-7.

## Introduction

With the wide use of low-dose computerized tomography (LDCT), the detection rate of lung malignant lesions appearing as ground-glass opacity nodules (GGOs) has risen rapidly [1–3]. Among the malignant GGOs detected, most of them are lung adenocarcinoma [4]. According to the pathological classification standard of World Health Organization (WHO), lung adenocarcinoma is classified into atypical adenomatous hyperplasia (AAH), adenocarcinoma in situ (AIS), minimally invasive adenocarcinomas (MIA) and invasive adenocarcinoma (IA) [5]. There is still no standard of intervention considering the invasiveness of lung adenocarcinoma manifesting as GGOs worldwide. Given that AAH and AIS nodules often grow slowly and considered of low-grade malignancy, clinical follow-up or sub-lobar lung resection are normally recommended and have yielded good response [6, 7]. Nodules confirmed as IA could grow fast and have risk of potential metastasis so that they are normally considered for lobectomy and lymphadenectomy. For nodules diagnosed as MIA, there has been controversy in surgical procedure selection [8, 9]. Nevertheless, a latest long-term follow-up study revealed that lobectomy was accomplished in 72.3% of patients with MIA, and recurrence was witnessed in patients with MIA (9.7%) but not in patients with AIS [10], indicating different intervention methods and prognosis for these two categories. Therefore, it is of great importance to distinguish preinvasive lesions (AAH and AIS) from invasive ones (MIA and IA), for it could potentially influence the treatment plan and follow-up schedule of the patients. However, although researchers have discovered that some variables such as maximum diameter of the nodule could be an indicator for the invasiveness of lung adenocarcinomas [11], it is still challenging to make a final diagnosis based on clinical and semantic CT features due to the overlap of morphological characteristics. Hence, it is necessary to explore models with better performance of predicting the invasiveness of lung adenocarcinoma appearing as GGOs.

Radiomics refers to the extraction and analysis of high-dimensional quantitative data derived from radiological images [12]. The segmentation of region of interest (ROI) is considered a crucial step in the process of radiomics. Because of the high contrast resolution between the pulmonary nodules and lung parenchyma which makes nodules easily delineated from adjacent lung tissue, radiomics was deemed as a suitable tool in the field of lung nodule assessment [13–15]. In some studies, radiomics method has been applied to predict the pathological subtype of lung adenocarcinoma appearing as GGOs, with area under the curve (AUC) of the model ranging from 0.62 to 0.89 [16–19]. However, these studies are limited by small sample size and few radiomics features. In addition, the diagnostic value of peripheral radiomic features of lung adenocarcinoma appearing as GGOs has not yet been fully explored. In the present study, we developed a nomogram using clinical, semantic CT characteristics and intra/peri-nodular radiomic features to differentiate preinvasive lesions from invasive lesions of lung adenocarcinoma manifesting as GGOs.

## Materials and methods

### Study cohort

**Fig. 1:**
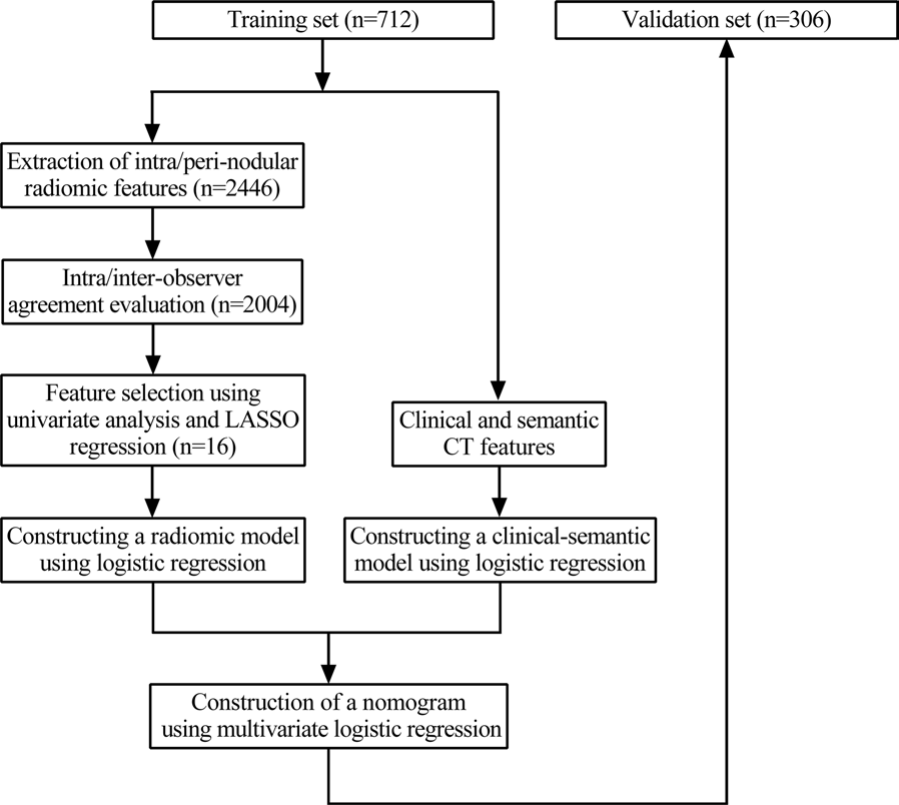
Flow chart of the study

From January 2018 to December 2019, patients diagnosed with lung adenocarcinoma manifesting as GGO and received lung resection in First Medical Center of Chinese People’s Liberation Army General Hospital was selected from the online medical record system to evaluate the eligibility for enrollment. In the present study, preinvasive lesions were defined as nodules that pathologically diagnosed as AAH or AIS, while invasive lesions were defined as nodules that diagnosed as MIA or IA. The clinical and radiographic data (listed in Table 1) were acquired and collected by reviewing the medical records. The inclusion criteria of the patients were as follows: (1) diagnosed as lung GGO on CT images and confirmed to be lung adenocarcinoma pathologically by lung resection; (2) maximum diameter of GGO was less than 30 mm; (3) had high-quality preoperative CT images with slice thickness less than 1.5 mm; (4) the interval between preoperative CT and lung resection was less than 2 weeks. Patients with CT image slice thickness greater than 1.5 mm, with incomplete clinical data or without specific pathological results were excluded. For multiple GGOs of the same patient, only those with confirmed pathological results were included in this study. The included lung nodules were then divided randomly into a training set and validation set with a ratio of 7:3, and the proportion of preinvasive lesions over invasive lesions in the two sets was kept to the same level (Fig. 1).

**Table 1:** Demographic, clinical and semantic CT features of patients in the training and validation set

Characteristic	Training set (n = 712)		p value	Validation set (n = 306)		p value
	Preinvasive lesions (n = 97)	Invasive lesions (n = 615)		Preinvasive lesions (n = 42)	Invasive lesions (n = 264)	
Gender						
Male	36 (37.1)	209 (34.0)	0.547	15 (35.7)	98 (37.1)	0.861
Female	61 (62.9)	406 (66.0)		27 (64.3)	166 (62.9)	
Age (years, average ± SD)	53.5 ± 8.5	54.9 ± 9.5	0.064	52.1 ± 10.9	55.1 ± 9.5	0.082
Having respiratory symptoms						
Yes	3 (3.1)	66 (10.7)	0.015	2 (4.8)	32 (12.1)	0.159
No	94 (96.9)	549 (89.3)		40 (95.2)	232 (87.9)	
BMI	24.1 ± 3.1	24.2 ± 3.0	0.761	23.4 ± 2.5	24.4 ± 3.0	0.07
Smoking history						
Yes	10 (10.3)	97 (15.8)	0.162	5 (11.9)	43 (16.3)	0.648
No	87 (89.7)	518 (84.2)		37 (88.1)	221 (83.7)	
Smoking index (pack-year)	87.6 ± 386.3	93.9 ± 283.0	0.177	100.0 ± 338.0	78.4 ± 226.6	0.544
Former lung cancer history						
Yes	2 (2.1)	14 (2.3)	1.000	0 (0)	2 (0.8)	1.000
No	95 (97.9)	601 (97.7)		42 (100)	262 (99.2)	
Former malignancy history except lung cancer						
Yes	4 (4.1)	33 (5.4)	0.806	0 (0)	11 (4.2)	0.372
No	93 (95.9)	582 (94.6)		42 (100)	253 (95.8)	
Former pulmonary benign disorders						
Yes	2 (2.1)	31 (5.0)	0.296	1 (2.4)	7 (2.7)	1.000
No	95 (97.9)	584 (95.0)		41 (97.6)	257 (97.3)	
Family history of lung cancer						
Yes	10 (10.3)	66 (10.7)	0.900	2 (4.8)	28 (10.6)	0.399
No	87 (89.7)	549 (89.3)		40 (95.2)	236 (89.4)	
Family history of malignancy except lung cancer						
Yes	17 (17.5)	93 (15.1)	0.543	7 (16.7)	41 (15.5)	0.851
No	80 (82.5)	522 (84.9)		35 (83.3)	223 (84.5)	
Abnormal tumor biomarker results^a^						
Yes	6 (6.2)	99 (16.1)	0.011	5 (11.9)	31 (11.7)	0.976
No	91 (93.8)	516 (83.9)		37 (88.1)	233 (88.3)	
Multiple nodules						
Yes	38 (39.2)	309 (50.2)	0.043	15 (35.7)	130 (49.2)	0.103
No	59 (60.8)	306 (49.8)		27 (64.3)	134 (50.8)	
Nodule density						
pGGO	81 (83.5)	374 (60.8)	< 0.001	35 (83.3)	163 (61.7)	0.007
mGGO	16 (16.5)	241 (39.2)		7 (16.7)	101 (38.3)	
Border						
Unclear	12 (12.4)	174 (28.3)	0.001	3 (7.1)	63 (23.9)	0.014
Clear	85 (87.6)	441 (71.7)		39 (92.9)	201 (76.1)	
Lobulation sign						
Yes	10 (10.3)	199 (32.4)	< 0.001	3 (7.1)	65 (24.6)	0.009
No	87 (89.7)	416 (67.6)		39 (9.3)	199 (75.4)	
Spiculation sign						
Yes	6 (6.2)	104 (16.9)	0.007	1 (2.4)	35 (13.3)	0.04
No	91 (93.8)	511 (83.1)		41 (97.6)	229 (86.7)	
Pleural indentation sign						
Yes	6 (6.2)	106 (17.2)	0.005	1 (2.4)	44 (16.7)	0.01
No	91 (93.8)	509 (82.8)		41 (97.6)	220 (83.3)	
Bubble sign						
Yes	8 (8.2)	107 (17.4)	0.023	4 (9.5)	40 (15.2)	0.478
No	89 (91.8)	508 (82.6)		38 (90.5)	224 (84.8)	
Vessel change						
Yes	14 (14.4)	125 (20.3)	0.174	4 (9.5)	64 (24.2)	0.044
No	83 (85.6)	490 (79.7)		38 (90.5)	200 (75.8)	
Maximum 2D diameter (mm, average ± SD)	9.2 ± 3.2	12.6 ± 5.2	< 0.001	8.9 ± 3.4	12.7 ± 5.1	< 0.001
Location						
Left upper lobe	20 (20.6)	152 (24.7)	0.277	12 (28.6)	69 (26.1)	0.670
Left lower lobe	21 (21.6)	91 (14.8)		7 (16.7)	34 (12.9)	
Right upper lobe	39 (40.2)	236 (38.4)		16 (38.1)	103 (39.0)	
Right middle lobe	2 (2.1)	33 (5.4)		3 (7.1)	12 (4.5)	
Right lower lobe	15 (15.5)	103 (16.7)		4 (9.5)	46 (17.4)	
Rad-score	0.9 ± 1.5	2.7 ± 1.3	< 0.001	1.1 ± 1.2	2.8 ± 1.3	< 0.001

BMI, body mass index; pGGO, pure ground-glass opacity nodule; mGGO, mixed ground-glass opacity nodule

^a^An abnormal tumor biomarker result is defined as a higher blood concentration above the normal range of any of the following: carcinoembryonic antigen (CEA), CA-125 or CYFRA21-1

### CT image acquisition, CT semantic features and building of a clinical-semantic model

All CT examinations were performed in one of the following scanners: Brilliance iCT (Phillips Medical Systems, Netherlands) and Somatom Definition (Siemens Medical Systems, Germany). The detailed information of the CT image acquisition was shown in Additional file 1: Table S1.

The CT images were reviewed by two physicians in the Department of Respiratory Diseases (M.Z and Z.Y with 5 years and 12 years of experience in lung CT imaging, respectively) who were blinded to the clinical profiles and pathological results of the patients. After reviewing the CT images, the physicians then evaluated the CT semantic characteristics in both the lung window (level: − 600HU, width: 1200HU) and the mediastinal window (level: 40HU, width: 400HU). If inconsistent results came up, a group meeting would be held to make a consensus. The collected CT semantic characteristics were listed in Table 1.

Univariate analysis was used to select significant clinical and semantic features on the training set. Multivariate logistic regression was then used to select independent factors and construct a clinical-semantic model. The performance of the constructed model was evaluated by calculating the AUC of the receiver operating characteristic (ROC) curve in both training and validation set.

### Segmentation, extraction of the radiomic features and intra/inter-observer agreement evaluation

**Fig. 2:**
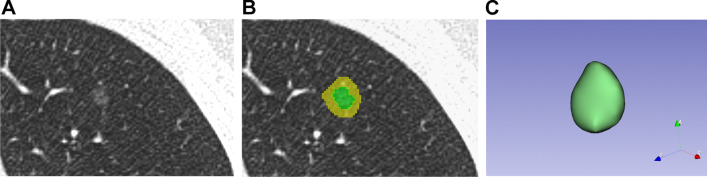
The segmentation of the regions of interest. **A** Computerized tomography (CT) image of a ground-glass opacity nodule pathologically confirmed as atypical adenomatous hyperplasia (AAH). **B** The segmentation of the nodule. Green area indicates the nodule region and yellow area indicates the peri-nodule region. **C** The constructed 3D model of the nodule in 3D slicer software

Intra/peri-nodular region segmentation was performed manually using 3D Slicer software (version 4.10.2, https://www.slicer.org). Intra-nodular region, defined as mask 1, was drawn on each slice of CT images to cover all nodule area by using function “Draw” in module “Segmentation” of 3D Slicer. Peri-nodular region, defined as mask 2, was obtained by extending mask 1 by 5 mm from its boarder in three dimensions using function “Hollow” in module “Segmentation” (Fig. 2).

Mask 1 and mask 2 were then used to extract the radiomic features using module “SlicerRadiomics extension” (http://pyradiomics.readthedocs.io/) in 3D Slicer. Firstly, the images were resampled to 1 mm × 1 mm × 1 mm to reduce the influence of different CT reconstruction method. Then a total of 1223 radiomic features were extracted in each mask based on the following five categories: (1) Shape; (2) First order features; (3) Texture features; (4) Wavelet features; (5) Laplacian of Gaussian (LoG) features. Finally, data of the radiomic features was standardized using method Z-score to eliminate the potential preference during the building of the radiomic model. Detailed list of radiomic features was presented in Additional file 1: Methods.

Intra-observer and inter-observer agreement were assessed by calculating intra/inter-class correlation coefficient (ICC). In order to evaluate the inter-observer agreement, two physicians (M.Z and Z.Y) performed the segmentation of the same 60 randomly selected lung nodules. Then 1 week later, M.Z finished the segmentation of the 60 lung nodules again to assess the intra-observer agreement. Both average ICC and ICC for each radiomic feature were calculated. Features with ICC < 0.75 were removed due to lack of stability. Because the two physicians showed good inter-observer agreement, M.Z alone finished the segmentation of the remaining nodules.

### Feature selection and building of a radiomic model

High dimensional data could lead to overfitting of the model, hence a dimensionality reduction of the radiomic features was essential. Here we used a two-step method for feature selection. First, Student’s t test or Mann–Whitney *U* test was applied to select significant features in the training set. Then least absolute shrinkage and selection operator (LASSO), a classic method to improve precision and reduce the possibility of model overfitting was used to select nonzero coefficient features. Tenfold cross-validation method was used to find the optimal regularization parameter (λ) in which the LASSO model had minimum error.

Next, the features selected by LASSO were used to construct a radiomic model in the training set. Logistic regression, support vector machines (SVM) and adaboost were used as the modeling method respectively, and the one with the best diagnostic performance was selected to build the final radiomic model. Radiomic-score (rad-score) was calculated according to the logistic regression model. The performance of the radiomic model was then confirmed in both training and validation set. Finally, the intra-nodular features and peri-nodular features were used to build two models respectively in order to compare their diagnostic value.

### Construction of a nomogram

Independent factors among the selected clinical, semantic features and rad-score were identified using a multivariate logistic regression. A diagnostic nomogram was then constructed based on the multivariate logistic regression model. A calibration curve was also plotted to evaluate the diagnostic efficiency of the nomogram.

### Clinical utility of the nomogram

Decision curve analysis (DCA) was performed to evaluate the clinical utility of the nomogram by calculating net benefits at different threshold probabilities.

### Statistical analysis

R software (version 4.0.2, The Free Software Foundation, USA) was used for statistical analysis, random grouping and model construction in this study. The “psych” package was used to calculate the ICC. The “glmnet” package was used to perform LASSO regression. The “e1071” package was used for SVM model construction. The “adabag” package was used to perform adaboost. The “pROC” package was used to plot the ROC curve. The “Hmisc”, “lattice”, “survival”, “Formula”, “ggplot2”, “rms” and “rmda” packages were used to construct the nomogram, calibration curve and decision curve.

Student’s t test, Pearson’s x^2^ test or Mann–Whitney *U* test was applied to evaluate the significance of clinical and semantic CT features, depending on the distribution and type of the data. Delong test was performed when comparing the AUCs of two ROC curves. A p value less than 0.05 was considered statistically significant.

## Results

### Baseline characteristics

From January 2018 to December 2019, a total of 1010 patients with 1018 GGOs were included in this study (As shown in Table 1). The GGOs were randomly divided into a training set (n = 712) and a validation set (n = 306). Among the 1018 lung nodules, 139 were preinvasive lesions (68 AAH and 71 AIS) and 879 were invasive lesions (145 MIA and 734 IA). In the preinvasive lesion group, 63.3% of the patients were female, and the average age was 53.0 ± 9.3 years. Only 5 patients (3.6%) suffered from respiratory symptoms, and most of the nodules were located in the right (39.6%) and left (23.0%) upper lobe. In the invasive lesion group, the average age of the patients was significantly higher (55.0 ± 9.5 years), and more patients had respiratory symptoms (11.1%), while the location of the nodules resembled that in the preinvasive lesion group. In the training set, there were significant differences in symptoms, abnormal tumor biomarker results, nodule amount (solitary vs multiple), nodule density, boarder (clear vs unclear), lobulation sign, spiculation sign, pleural indentation sign, bubble sign and maximum 2D diameter between the preinvasive lesion group and invasive lesion group. A multivariate logistic regression showed that abnormal tumor biomarker results, nodule density, lobulation sign and maximum 2D diameter were independent factors associated with the invasiveness of lung adenocarcinoma appearing as GGOs (odds ratio: 2.419, 1.926, 2.711 and 1.180, respectively), which were then used to construct a clinical-semantic model by logistic regression. The AUCs of the clinical-semantic model in training set and validation set were 0.755 (95% CI 0.707–0.804) and 0.746 (95% CI 0.667–0.824), respectively.

### Intra-observer and inter-observer agreement

For intra-observer agreement evaluation, the average ICC of the total radiomic features (n = 2446, including intra-nodule and inter-nodule features) was 0.90, and the ICCs of 2181 features were higher than 0.75. For inter-observer agreement evaluation, the average ICC was 0.89, and an ICC higher than 0.75 was observed in 2122 features (Additional file 1: Fig. S1). In total, 442 features that had an ICC lower than 0.75 were removed. The results showed that the 2 observers showed good agreement in nodule segmentation and feature extraction, and the remaining 2004 robust radiomic features were used for feature selection and model construction.

### Feature selection, performance of the radiomic model and diagnostic value of peri-nodular features

**Fig. 3:**
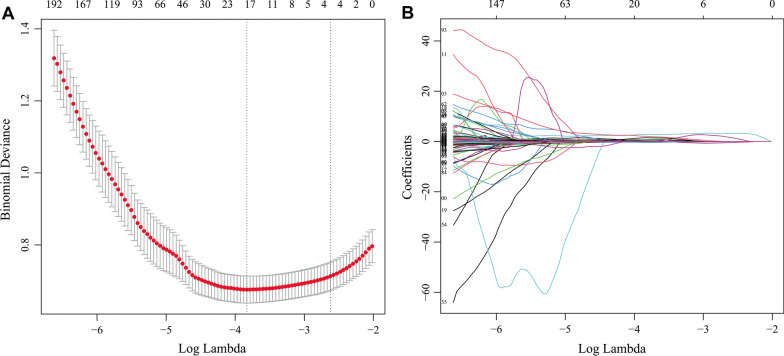
Feature selection using least absolute shrinkage and selection operator (LASSO). **A** Tenfold cross-validation analysis of LASSO was performed and when λ = 0.022, logλ = − 3.817 (the first dotted vertical line), the model had minimum error, and 16 non-zero features were selected. **B** The coefficient profiles of the 1789 features

**Fig. 4:**
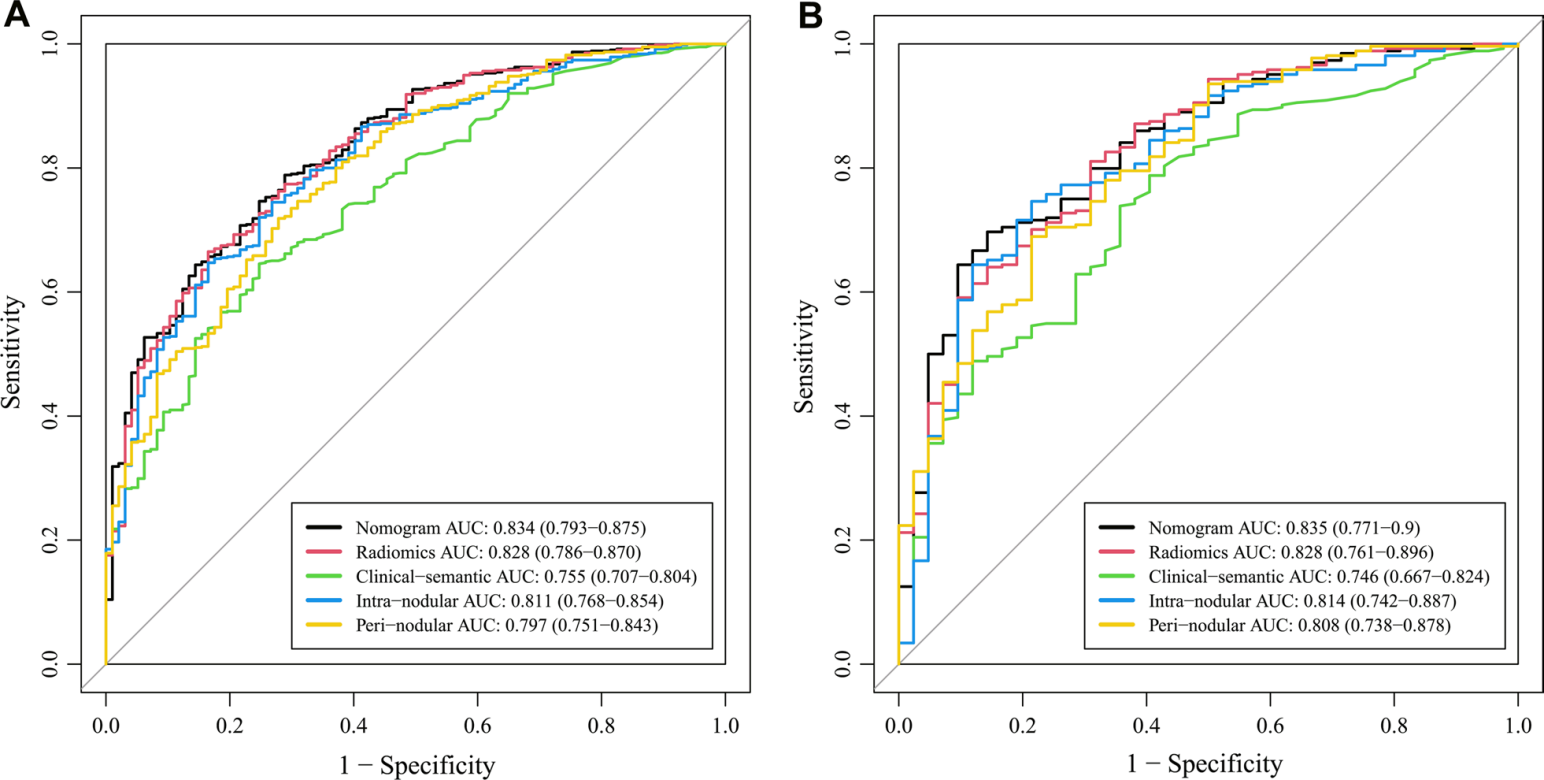
The receiver operating characteristic (ROC) curves showing the performance of the nomogram, radiomic model, clinical-semantic model, intra-nodular radiomic model and peri-nodular radiomic model in **A** training and **B** validation set. A Delong test showed that the nomogram exhibited better performance comparing to the clinical-semantic model in both training (p = 0.0002) and validation set (p = 0.003). Data in the parentheses referred to the 95% confidence interval of area under the curve (AUC)

A univariate analysis showed that 1789 out of 2004 radiomic features were significantly different between the preinvasive lesion group and invasive lesion group in the training set (data not shown). Then LASSO regression was performed and as shown in Fig. 3A, when λ = 0.022, logλ = − 3.817, the model had the minimum error, and 16 nonzero features were selected and used for construction of a radiomic model. Among the three methods used for modeling, logistic regression was found to have the best performance in the validation set (Additional file 1: Fig. S2), which was then used to build the radiomic model. Rad-score was calculated according to the results of the radiomic model, and it was significantly higher in the invasive lesion group than preinvasive lesion group in both training set (2.7 ± 1.3 vs 0.9 ± 1.5, p < 0.001) and validation set (2.8 ± 1.3 vs 1.1 ± 1.2, p < 0.001). The formula of rad-score was presented in Additional file 1: Methods. As shown in Fig. 4, the AUCs of the radiomic model were significantly higher than those of the clinical-semantic model in both training set (0.828 vs 0.755, p = 0.001) and validation set (0.828 vs 0.746, p = 0.008), indicating that a better diagnostic efficiency was observed when using radiomic features rather than clinical and semantic CT features.

Among the selected features, there were nine intra-nodular features and seven peri-nodular ones, which signified that features surrounding the nodule were also highly informative and important. To explore the diagnostic value of peri-nodular features in specific, intra-nodular features and peri-nodular ones were separated and used to build two models based on logistic regression. The AUCs for intra-nodular model and peri-nodular model were 0.814 and 0.808 in the validation set, and a combination of the models raised the AUC to 0.828 (Fig. 4). These results showed that peri-nodular features were also good predictors for lung adenocarcinoma invasion evaluation.

### Nomogram and clinical utility

**Fig. 5:**
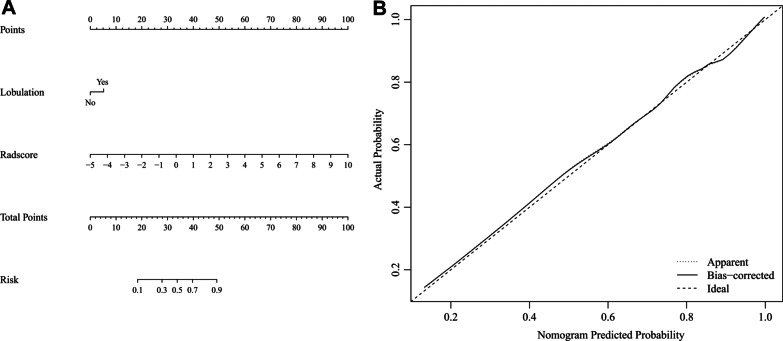
The nomogram and calibration curve. **A** The constructed nomogram based on lobulation sign and rad-score. **B** The calibration curve of the established nomogram. The curve showed that the nomogram had a good agreement between prediction and observation

Multivariate logistic regression of the selected clinical-semantic features (abnormal tumor biomarker results, nodule density, lobulation sign and maximum 2D diameter) and rad-score revealed that lobulation sign and rad-score was independent factors associated with the invasiveness of lung adenocarcinoma appearing as GGOs (Table 2). These two variables were then used for the construction of a diagnostic nomogram (Fig. 5). The nomogram exhibited the best discrimination ability comparing with the radiomic model and clinical-semantic model in both the training set (AUC = 0.834) and the validation set (AUC = 0.835).

**Table 2:** Results of multivariate logistic regression of significant features

Variables	β	OR (95% CI)	p value
Intercept	0.049		0.89
Lobulation	0.76	2.138 (1.029–4.442)	0.042
Rad-score	0.976	2.653 (2.058–3.422)	< 0.001

β, regression coefficient; OR, odds ratio; CI, confidence interval

**Fig. 6:**
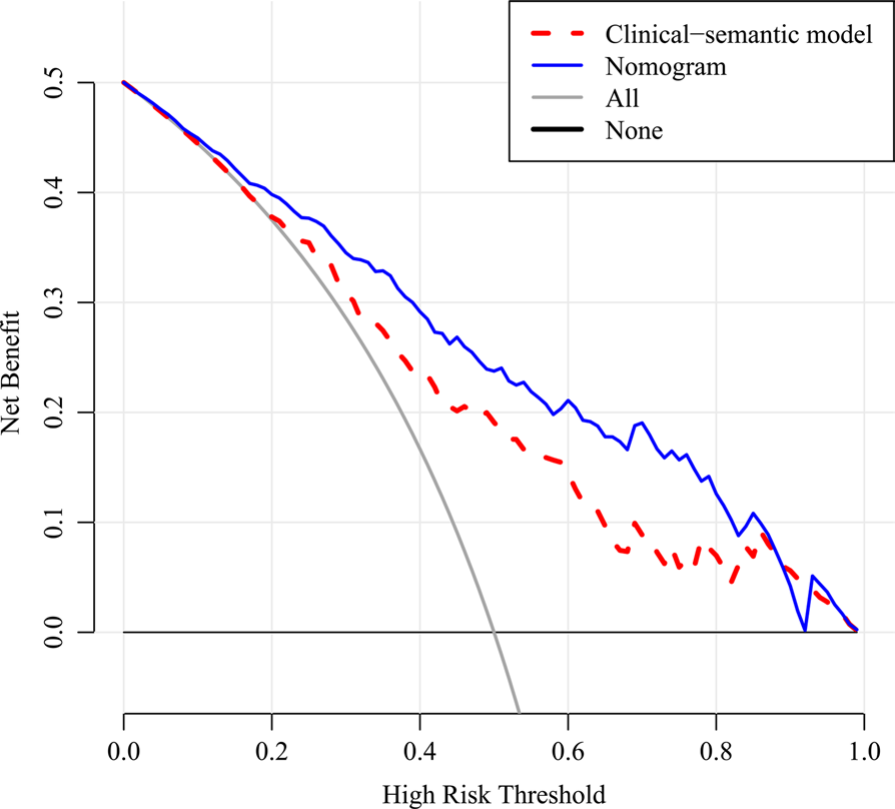
Decision curve analysis (DCA) of the nomogram and the clinical-semantic model. The DCA curve showed that within the threshold probability ranging from 10 to 90%, using the nomogram added more net benefits than clinical and semantic CT features in differentiating preinvasive lung adenocarcinoma manifesting as ground-glass opacity nodules from invasive ones

DCA was performed (Fig. 6) and the results showed that within the threshold probability ranging from 10 to 90%, using the nomogram added more net benefits than clinical and semantic CT features in differentiating preinvasive lung adenocarcinoma manifesting as GGOs from invasive ones.

## Discussion

The present study aimed to evaluate the performance and clinical value of radiomic features to assess the invasiveness of lung adenocarcinoma manifesting as GGOs. A total of 1018 GGOs with 2446 intra-/peri-nodular radiomic features and 22 clinical and semantic CT features were included in this study. After feature selection and model construction process, the established radiomic-based nomogram exhibited better diagnostic efficiency and clinical value than using clinical and semantic CT features alone.

At present, clinical and semantic CT features are commonly applied to recognize an invasive lesion appearing as GGO. Previous studies have explored their clinical value. A study involving 272 GGOs during a 6-year span showed that large nodule maximum diameter (with cut-off of 10 mm), lobulated boarder and spiculated margin were predictors for invasiveness of lung adenocarcinoma appearing as mixed GGOs [11], while some researchers [20] found that GGOs with size larger than 16.4 mm were more likely to be invasive lesions. In our study, nodule maximum diameter, nodule density and lobulation sign were also found to be related to nodule invasiveness. Moreover, we discovered that abnormal lung cancer related tumor biomarker results could be a predictor for nodule invasiveness, which was seldom involved in previous studies. However, in our study, the combination of clinical and semantic CT features only exhibited moderate performance (AUC = 0.746), and mistakenly diagnosing an invasive lesion into a preinvasive one is risky cause it could lead to shortened survival of the patients. Therefore, we explored the potential of radiomic features and try to obtain a model with better performance.

With high dimensional data, radiomics is a potentially more valuable method for nodule invasiveness evaluation comparing with clinical and semantic features, for it could recognize and extract the tiny changes that are unnoticeable with naked eyes. In a retrospective study, researchers used five radiomic features to construct a radiomic model for nodule invasiveness prediction, which yielded good efficiency (AUC = 0.89) [16]. Another study showed that a radiomic-based model using two features exhibited excellent performance (AUC = 0.942 on a validation cohort) [21]. In our study, 16 robust radiomic features were selected and used to construct a radiomic model, which showed good predicting ability for lung adenocarcinoma invasion with AUC of 0.828. Different from other researches, the selected radiomic features in our study were all extracted from wavelet and LoG filtered images. These filters could strengthen certain characteristics of the original image to reveal some information that was hidden before. These results showed that image filtering could dig out more information from CT slices and should be used more frequently in radiomic researches. In addition, rad-score and lobulation sign were selected as independent factors for nodule invasion prediction and were used to construct a nomogram. The nomogram showed best performance with an AUC of 0.835, and a decision curve analysis revealed that it had better clinical value than using clinical and semantic CT features alone. With the nomogram, the quantitative risk for invasiveness of a certain GGO could be calculated precisely, which could then be used as a reference to assist the judgement of clinicians in processes of nodule intervention and management.

It is known that invasive lung adenocarcinoma could have influence on the surrounding environment such as the form of micro-vessels. Moreover, in some peri-nodular area, there might be actual differences but unnoticeable on CT slices, which might give rise to changes on a radiomic scale. Hence, peri-nodular features could have potential values in radiomic researches [22, 23]. A few studies [24, 25] have explored the value of peri-nodular features in distinguishing preinvasive lesions from invasive ones, however, these studies were limited by their sample size and radiomic feature number. Therefore, we evaluated the performance of peri-nodular features extracted from the 5 mm ring area surrounding the nodule in our study. The results signified that a radiomic model based on pure peri-nodular area was useful in differentiating preinvasive lesions from invasive ones with an AUC of 0.808, and a combination of both intra-nodular and peri-nodular features exhibited best performance. This shows that nodule surroundings are also important and should be paid more attention to in future radiomic researches.

In some studies, radiomic methods were found to have relatively unsatisfactory performance. Luo et al. [18] found that a radiomic model had an AUC of 0.769, comparing to 0.853 of a clinical model. In another study, both radiomic features and clinical features exhibited poor performance in the testing cohort when used to predict nodule invasiveness [17]. Although performance of radiomic features changes with distribution of the data, it is also greatly related to dimension of the features and modeling methods. According to our results, we tried three different modeling methods and the performance varied from 0.677 to 0.828 in the validation set, which highlighted the importance of selecting a suitable modeling method. A study [26] involving 12 different modeling methods also found that with different classifiers, the variance of AUC of the established models could be as high as 40%. In addition, the same classifier might have different performance in various of data sets. Therefore, to extend the full potential of radiomics, incorporating more radiomic features and trying more modeling methods are encouraged to obtain the radiomic model with the best performance.

This study has several limitations. First, in order to ensure a confirmed pathological result, this study only included patients who received lung resection. This inclusion criteria ruled out the patients who were unwilling to take the operation or those who were in a follow-up process, which might cause a selection bias. Second, external validation was not accomplished in this single-center retrospective study, which could lead to bias of the model performance. Third, quantitative CT features, such as nodule mass, solid proportion and pleural contact index have been reported to be useful in nodule assessment [27–29], which were not included in this study. The combination of quantitative CT features with radiomic features could be of clinical value. Lastly, due to difficulties in data collection, longitudinal follow-up CT data of patients was not included in this study. The radiomic model based on these data could be used to indicate the turning point when a preinvasive lesion becomes an invasive one, which might help to shorten the follow-up period of the patients. Therefore, future studies are still needed to break through the above limitations.

## Conclusions

Radiomic model showed a better performance in assessing the invasiveness of lung adenocarcinoma appearing as GGOs than clinical and semantic CT model. Besides intra-nodular radiomic features, peri-nodular radiomic features also played an important part in nodule invasive assessment. A nomogram incorporated clinical, semantic and radiomic features showed good performance and clinical value, which could provide more information for clinicians in the process of nodule evaluation, intervention and management.

## Supplementary Information

**Additional file 1. **Additional methods, Additional tables and Additional figures.

## Data Availability

The datasets generated during the current study are not publicly available due to our institutional regulation, but are available from the corresponding author on reasonable request.

## Abbreviations

CT: Computerized tomography
LDCT: Low-dose computerized tomography
GGO: Ground-glass opacity nodule
AAH: Atypical adenomatous hyperplasia
AIS: Adenocarcinoma in situ
MIA: Minimally invasive adenocarcinomas
IA: Invasive adenocarcinoma
ROI: Region of interest
AUC: Area under the curve
ROC: Receiver operating characteristic
LoG: Laplacian of Gaussian
ICC: Intra/inter-class correlation coefficient
LASSO: Least absolute shrinkage and selection operator
SVM: Support vector machines
rad-score: Radiomic score
DCA: Decision curve analysis
